# Case report of sigmoid colon cancer with synchronous peritoneal metastasis achieving a pathological complete response after mFOLFOX6 plus cetuximab

**DOI:** 10.1016/j.ijscr.2025.112022

**Published:** 2025-10-07

**Authors:** Kohei Takehara, Hiroaki Kasashima, Tatsunari Fukuoka, Yuki Seki, Masatsune Shibutani, Kiyoshi Maeda

**Affiliations:** Department of Gastroenterological Surgery, Osaka Metropolitan University Graduate School of Medicine 1-4-3 Asahimachi, Abeno-ku, Osaka, 545-8585, Japan

**Keywords:** Colorectal cancer, Synchronous peritoneal metastasis, Synchronous colorectal cancer, Pathological complete response, Case report

## Abstract

**Introduction:**

Synchronous peritoneal metastasis occurs in about 5 % of colorectal cancer (CRC) cases, often with other distant metastases. Due to poor drug penetration, chemotherapy is typically ineffective, leading to poor prognosis. Here, we report a rare case of sigmoid colon cancer with synchronous peritoneal metastasis achieving pathological complete response (pCR) after systemic chemotherapy.

**Case presentation:**

A 74-year-old man was diagnosed with acute generalized peritonitis from a perforated sigmoid colon cancer with two peritoneal metastases. One peritoneal lesion was left unresected, and sigmoid colectomy with partial jejunal resection was performed. Additionally, postoperative colonoscopy revealed a synchronous cancer in the cecum. Chemotherapy with mFOLFOX6 plus cetuximab was initiated. After 10 cycles, the patient developed strangulation ileus, requiring surgery, during which a peritoneal nodule was resected and showed no viable cancer cells, indicating pCR. Chemotherapy was resumed and completed to 12 cycles. Laparoscopic ileocecal resection for the cecal cancer also achieved pCR. A single peritoneal recurrence was surgically removed. No recurrence has been observed for 7 months.

**Discussion:**

Reports of pCR with peritoneal metastases are extremely rare. Although the mechanism of the remarkable response in this case remains unclear, given the rarity of pCR in CRC with peritoneal metastasis, further case accumulation and analysis are warranted.

**Conclusion:**

This is a rare case of pCR to systemic chemotherapy for CRC with synchronous peritoneal metastasis.

## Highlights

• A rare case of synchronous peritoneal metastasis from colorectal cancer achieving pathological complete response after mFOLFOX6 plus cetuximab.
• The case highlights remarkable chemotherapy efficacy even with poor drug penetration into the peritoneum.
• This outcome is particularly noteworthy and represents an extremely rare clinical entity, contributing to the accumulation of knowledge in this field.

## 1 Introduction

Synchronous peritoneal carcinomatosis occurs in approximately 5 % of patients with colorectal cancer (CRC) [1,2], and in the majority of such cases, other distant metastases are also present [3,4]. Pathological complete response (pCR) following chemotherapy for CRC is extremely uncommon [5,6]. Cases with peritoneal dissemination are particularly resistant to chemotherapy, as drug penetration into the peritoneum is poor [7,8]. Here, we report a case of sigmoid colon cancer with synchronous peritoneal metastasis that achieved a pCR following systemic chemotherapy. This case report has been reported in line with the SCARE checklist [9].

## 2 Case presentation

**Fig. 1:**
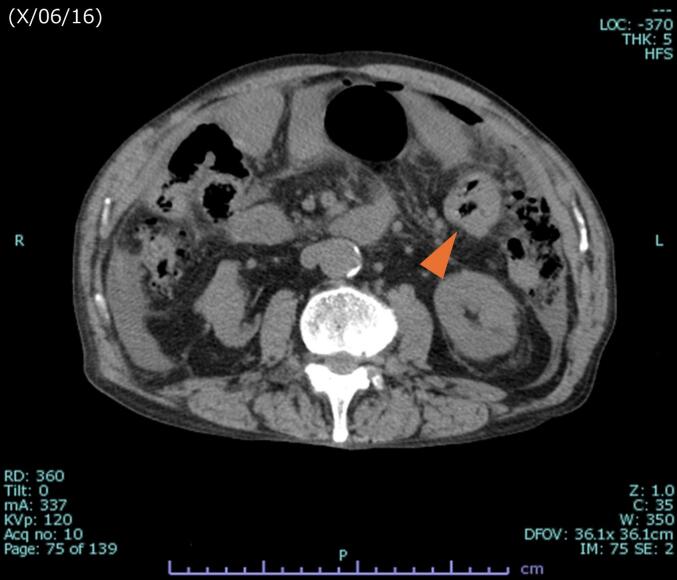
CT imaging at admission. Contrast-enhanced CT revealed thickening of the sigmoid colon wall, increased density of the surrounding fatty tissue, and the presence of free intraperitoneal air.

A 74-year-old man was transferred to our hospital for emergency laparotomy due to sigmoid colon perforation and generalized peritonitis. On arrival, his abdomen was board-like rigid. Contrast-enhanced computed tomography (CT) revealed thickening of the sigmoid colon wall, increased density of the surrounding fatty tissue, and the presence of free intraperitoneal air (Fig. 1). Positron emission tomography-computed tomography (PET-CT) was not performed at the time of admission. He had no notable medical history, medication use, or family history of colorectal or other hereditary cancers. An emergency sigmoid colectomy with D1 lymph node dissection and colostomy was performed. Intraoperatively, two peritoneal dissemination nodules were observed: one on the abdominal wall just above the peritoneal reflection and one on the jejunal mesentery. The abdominal wall lesion was incompletely resected, with only partial removal achieved. Partial resection of the jejunum was performed for the latter lesion.

Postoperative pathological examination revealed that the sigmoid colon cancer was a moderately differentiated tubular adenocarcinoma (tub2), pT3N1aM1, pStage IV. Surgical margins were negative, and three of nine retrieved lymph nodes showed metastatic involvement. The nodule on the jejunal mesentery was also diagnosed as metastatic adenocarcinoma. Molecular analysis showed no mutations in RAS, BRAF or microsatellite instability (MSI).

Additionally, postoperative colonoscopy revealed a type 3 tumor in the cecum, leading to a diagnosis of synchronous cecal cancer. Pathological findings from the biopsy specimens confirmed a moderately differentiated tubular adenocarcinoma (tub2) without BRAF mutation or MSI.

Based on these findings, the patient was diagnosed with perforated sigmoid colon cancer with synchronous peritoneal carcinomatosis and synchronous cecal cancer. He received chemotherapy with mFOLFOX6 plus cetuximab (Cmab). As endoscopic findings suggested low risk of immediate obstruction from the cecal tumor, systemic chemotherapy was prioritized, with surgery for cecal cancer planned afterward.

**Fig. 2:**
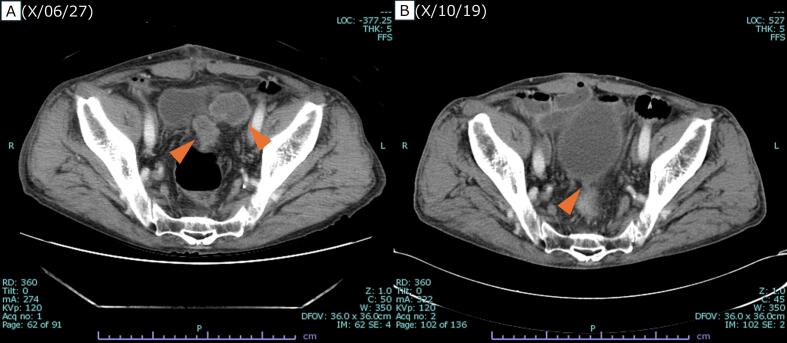
Contrast-enhanced CT after five courses of chemotherapy. Significant reduction in the size of the peritoneal nodules was observed following five cycles of mFOLFOX6 plus cetuximab. A, Before initiation of chemotherapy. B, After five courses of chemotherapy.

**Fig. 3:**
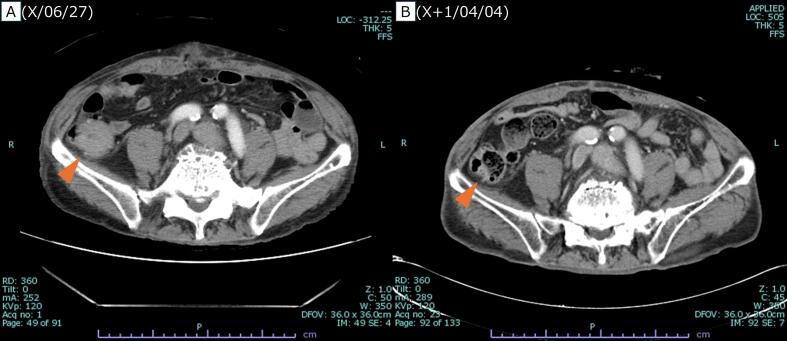
Preoperative CT for ileocecal resection. Contrast-enhanced CT showed shrinkage of the cecal cancer after systemic chemotherapy. A, Pre-chemotherapy. B, Post-chemotherapy.

**Fig. 4:**
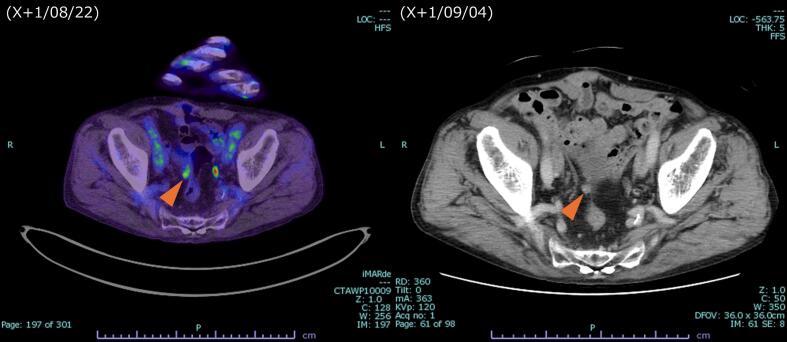
Recurrent peritoneal nodule. PET-CT demonstrated FDG accumulation (SUVmax: 3.8) at the anterior rectal wall, indicating recurrent peritoneal metastasis.

12 cycles of mFOLFOX6 plus Cmab were initiated the following month. 2 months after onset, after 5 cycles, contrast-enhanced CT showed shrinkage of the peritoneal nodules (Fig. 2). 7 months after onset, after 10 cycles, the patient developed strangulation ileus and underwent emergency laparotomy. Intraoperatively, a peritoneal nodule was found in the Douglas pouch and resected. Pathological examination revealed no viable cancer cells, indicating a pCR. Chemotherapy was resumed and completed up to 12 cycles. Subsequently, 9 months after onset, contrast-enhanced CT showed no detectable peritoneal metastasis, and PET-CT showed no significant fluorodeoxyglucose (FDG) uptake. 10 months after onset, laparoscopic ileocecal resection with D3 lymph node dissection was performed for the cecal cancer (Fig. 3), and pathological examination again revealed pCR. However, 4 months later, follow-up PET-CT detected a recurrent peritoneal nodule (Fig. 4). 2 months later, during surgery for colostomy closure, the recurrent nodule was resected. Pathological analysis confirmed recurrent adenocarcinoma. The patient has been under follow-up without recurrence for 7 months.

## 3 Discussion

Synchronous peritoneal dissemination is observed in approximately 5 % of patients with colorectal cancer (CRC) [1,2], and the majority (about 55 %) of these cases involve other distant organ metastasis as well [3,4]. Meanwhile, synchronous colorectal cancer has been reported to occur in approximately 1–8 % of all CRC cases [10], with the most common sites being the sigmoid colon, rectum and cecum/appendix, in that order [11]. Thus, the present case, involving both synchronous peritoneal dissemination and synchronous multiple CRC, represents an extremely rare clinical entity.

Furthermore, complete response (CR) to chemotherapy in unresectable CRC is exceedingly rare. The CR rate with FOLFOX6 as a first-line treatment for metastatic CRC is about 5 % [5], and with FOLFOX4 for CRC with liver metastases, the CR rate is about 3 % [6]. Reports of pathological complete response (pCR) after chemotherapy in CRC with peritoneal metastases are even more limited. In this case, pCR was achieved for both peritoneal metastasis and synchronous cecal cancer following systemic chemotherapy, making it particularly noteworthy. The chemotherapy regimen, consisting of mFOLFOX6 plus cetuximab, was selected in consultation with an experienced medical oncologist, considering the tumor's RAS/BRAF wild-type status and the need for a prompt and effective systemic response.

This multidisciplinary approach contributed to the favorable outcome observed in this case.

For the treatment of colorectal cancer with peritoneal dissemination, surgical resection, systemic chemotherapy, and hyperthermic intraperitoneal chemotherapy (HIPEC) are generally considered. In this case, HIPEC was not pursued owing to the emergency presentation and the absence of a definitive indication at that time. Instead, a sigmoid colectomy aimed at life-saving intervention was performed, followed by postoperative systemic chemotherapy.

The surgical procedure performed for this patient is described below. Upon presentation, the patient had already developed generalized peritonitis, and life-saving management was prioritized over achieving an oncologically adequate resection. As a result, a full lymphadenectomy was not performed; instead, a limited D1 dissection was undertaken. The lesion on the abdominal wall was located cranial to the peritoneal reflection and thus outside the operative field during the emergency laparotomy. To obtain sufficient exposure, adhesiolysis between the bowel and the abdominal wall was performed, and the lesion was partially resected as much as possible.

Concerning the synchronous cecal cancer, it was not resected during the initial laparotomy. This was due to the absence of acute obstruction and the critically ill condition of the patient, necessitating prioritization of life-preserving measures. For the surgical management of the cecal cancer following systemic chemotherapy, a segmental colectomy was chosen over a subtotal colectomy, taking into consideration the patient's age, overall clinical status, and expected postoperative quality of life.

With regard to genetic analyses, microsatellite instability (MSI) and somatic mutation testing for RAS and BRAF were performed; however, germline testing was not conducted. In patients presenting with multiple primary colorectal cancers, Lynch syndrome should be considered as a potential differential diagnosis. In this case, the absence of a personal or family history suggestive of hereditary cancer syndromes made the likelihood of Lynch syndrome low. Nonetheless, further genetic evaluation may be warranted if additional clinical or familial information emerges during follow-up.

For follow-up assessment, CT, PET-CT, and tumor markers (CEA, CA19-9) were utilized. Regarding the FDG sensitivity of the tumor, uptake of FDG was observed at the time of recurrence in the abdominal wall nodule, indicating that the tumor was FDG-avid. Therefore, PET-CT was considered a useful modality for follow-up in this case.

The recurrence is described in the following section. Although pCR was achieved in the sigmoid colon cancer, the peritoneal lesion, and the cecal tumor, a recurrence was detected during follow-up. It remains possible that the peritoneal lesion resected during emergency surgery for strangulation ileus had responded completely, while microscopic residual disease remained elsewhere. However, the absence of visible metastases on post-chemotherapy contrast-enhanced CT and PET-CT, combined with the pCR of the resected cecal cancer, supports the conclusion that a complete response had indeed been achieved at that point.

Regarding post-recurrence management, additional chemotherapy was not administered. Considering the initial favorable response to chemotherapy, we opted for close surveillance with CT every three months rather than immediate re-treatment. This underscores the importance of vigilant long-term follow-up, given the possibility of late recurrence even after pCR. If no recurrence is observed within a short period, the intervals of CT imaging was planned to be progressively lengthened, and long-term follow-up is continued. The timing of conversion surgery after chemotherapy for peritoneal metastases remains controversial. In this case, emergency surgery for strangulation ileus serendipitously provided an opportunity to obtain pathological confirmation of the treatment response.

The precise reason for the remarkable chemotherapy efficacy in this case remains unclear. However, previous reports have demonstrated that oxaliplatin combined with targeted agents significantly improves the prognosis in CRC patients with peritoneal metastasis [12]. This case may represent another instance where such a treatment strategy was successful. Given the rarity of pCR in CRC with peritoneal metastasis, further case accumulation and analysis are warranted. To our knowledge, there have been no previous reports describing pathological complete response to systemic chemotherapy in a patient with synchronous double primary colorectal cancers (sigmoid and cecal), accompanied by synchronous peritoneal metastasis. This case may thus represent an exceptional and clinically informative scenario. In the future, the development of immune checkpoint inhibitors and other novel agents may further improve treatment outcomes for this challenging condition.

## 4 Conclusion

This case represents a rare instance of pathological complete response to systemic chemotherapy for colorectal cancer with synchronous peritoneal metastasis.

## Consent

Written informed consent was obtained from the patient for publication of this case report and any accompanying images. A copy of the written consent is available for review by the Editor-in-Chief of this journal on request.

## Ethical approval

The procedures adhered to the tenets of the Declaration of Helsinki. This research is being conducted with the approvalof the Ethics Committee.

## Guarantor

Hiroaki Kasashima

## Sources of funding

There are no sources of funding to declare.

## Author contribution

Writing – original draft: Kohei Takehara

Supervision: Hiroaki Kasashima, Tatsunari Fukuoka

Writing – review & editing: Yuki Seki, Masatsune Shibutani, Kiyoshi Maeda

## Declaration of competing interest

The authors declare that they have no conflicts of interests.
